# Influence of different patient positions on erector spinae plane block spread in modified radical mastectomy: a prospective randomized comparative study

**DOI:** 10.1186/s12871-025-03231-7

**Published:** 2025-07-30

**Authors:** Adel Ibrahim Hozien, Sahar Ahmed Elkaradawy, Hussein M. Agameya, Walid M. Ahmed, Ahmed F. Elsosy

**Affiliations:** 1 Department of Anesthesia and Pain Management, Medical Research Institute, Alexandria University, Alexandria, Egypt; 2 Department of Anesthesia and Postoperative Intensive Care, Alexandria Faculty of Medicine, Alexandria University, Alexandria, Egypt; 3 Department of Diagnostic Radiology, Medical Research Institute, Alexandria University, Alexandria, Egypt

**Keywords:** Erector spinae plane block, Regional anesthesia for mastectomy, Postoperative analgesia, Computed Tomography, Patient position and local anesthetic spread, Opioid consumption

## Abstract

**Background:**

Although the analgesic effect of erector spinae plane block (ESPB) has been proven, its efficacy may be influenced by the patient’s position during the block.

**Methods:**

We randomly allocated forty patients undergoing unilateral modified radical mastectomy (MRM) with axillary lymph node dissection to receive preoperative ESPB in sitting (Group 1) or lateral position (Group 2), and the patient was kept in position for 15 min, either in the high Fowler’s or lateral position. The primary outcome was the dermatomal sensory block and radiocontrast material spread by Computed Tomography (CT) 15 min after the block. Secondary outcomes were the visual analog scale for pain (VAS), opioid consumption, patient satisfaction, and complications.

**Results:**

There was a significant increase in the dermatomal sensory block in Group 1 compared to Group 2 at midaxillary (confidence interval (C.I) 95% = 0.55, *p*-value = 0.006) and scapular lines (95% C.I **=** 0.50, *p*-value = 0.014); meanwhile, there were insignificant differences at the parasternal and midclavicular lines (*p*-value = 0.232 and 0.201 respectively). Early CT showed more craniocaudal contrast distribution in a higher percentage of patients in Group 1, with a higher incidence of spread to paravertebral/epidural spaces (though not statistically significant). The VAS, morphine consumption, and patient satisfaction were comparable.

**Conclusion:**

The ESPB in the sitting position provided a more significant posterolateral sensory block. The CT evidence of early paravertebral and epidural spread was observed more often in the sitting group, but without statistical significance. ESPB in both patient positions produced comparable postoperative analgesia.

**Trial registration:**

Pan African Clinical Trials Registry (PACTR) (PACTR202204720116048) on 27/04/2022.

## Introduction

Breast cancer is the world’s fifth most common cause of mortality [1]. Postoperative pain after mastectomy is a combination of nociceptive and neuropathic pain that, if untreated, can progress to chronic pain and have a detrimental influence on quality of life [2]. Erector spinae plane block (ESPB) is an interfacial plane block first published in 2016 by Forero et al. [3] as a successful modality for controlling thoracic nociceptive and neuropathic pain. Its efficiency is influenced by the compartmental spread and distribution of local anesthetic (LA) to neighboring targeted nerves. Many previous studies revealed that the injectate in ESPB could extend to the dorsal and ventral rami of the spinal nerve cells, blocking somatic and visceral pain [4, 5].

Most studies on the spread of local anesthetics in ESPB were cadaveric. Unfortunately, the findings of these experiments were contradictory, as not all showed considerable dye diffusion [6, 7]. A cadaveric model’s dye dispersion may differ from that of living persons. Anatomical variables that may alter the spread include differences in tissue tension, body temperature, muscle tone, injectate density, and changes in intrathoracic pressure caused by respiration, which are not present in cadavers [8].

Other factors that could influence LA distribution after ESPB are LA volume, unilateral or bilateral block, level of local anesthetic injection, and patient position. ESPB can be administered in sitting, prone, or lateral decubitus positions.

We hypothesized that performing ESPB in two different patient positions (sitting vs. lateral) with the patient kept in position for 15 min either in the lateral or high Fowler’s position may affect the early spread of ESPB measured by ipsilateral dermatomal sensory block and distribution of radiocontrast material spread underneath the erector spinae plane, paravertebral and epidural spaces as evidenced by Computed Tomography (CT) and thereby affect analgesic outcomes.

This study’s scientific and clinical significance lies in identifying the optimum patient position and its potential effect on early block spread and, subsequently, the onset and efficacy of analgesia. It offers scientific evidence for clinicians to investigate and select preferred patient postures during regional blocks, which could be applied in various positions. Thus optimizing pain management strategies and improving patients’ recovery and quality of life.

## Patients and methods

This prospective randomized comparative trial was registered on the Pan African Clinical Trials Registry (PACTR) on 28 April 2022 (PACTR202204720116048) after obtaining approval from the Faculty of Medicine Ethical Committee (IRB No: 00012098 FWA No: 00018699 Serial Number: 0106867). The study follows the Consolidated Standards of Reporting Trials (CONSORT) 2010 statement [9].

**Fig. 1:**
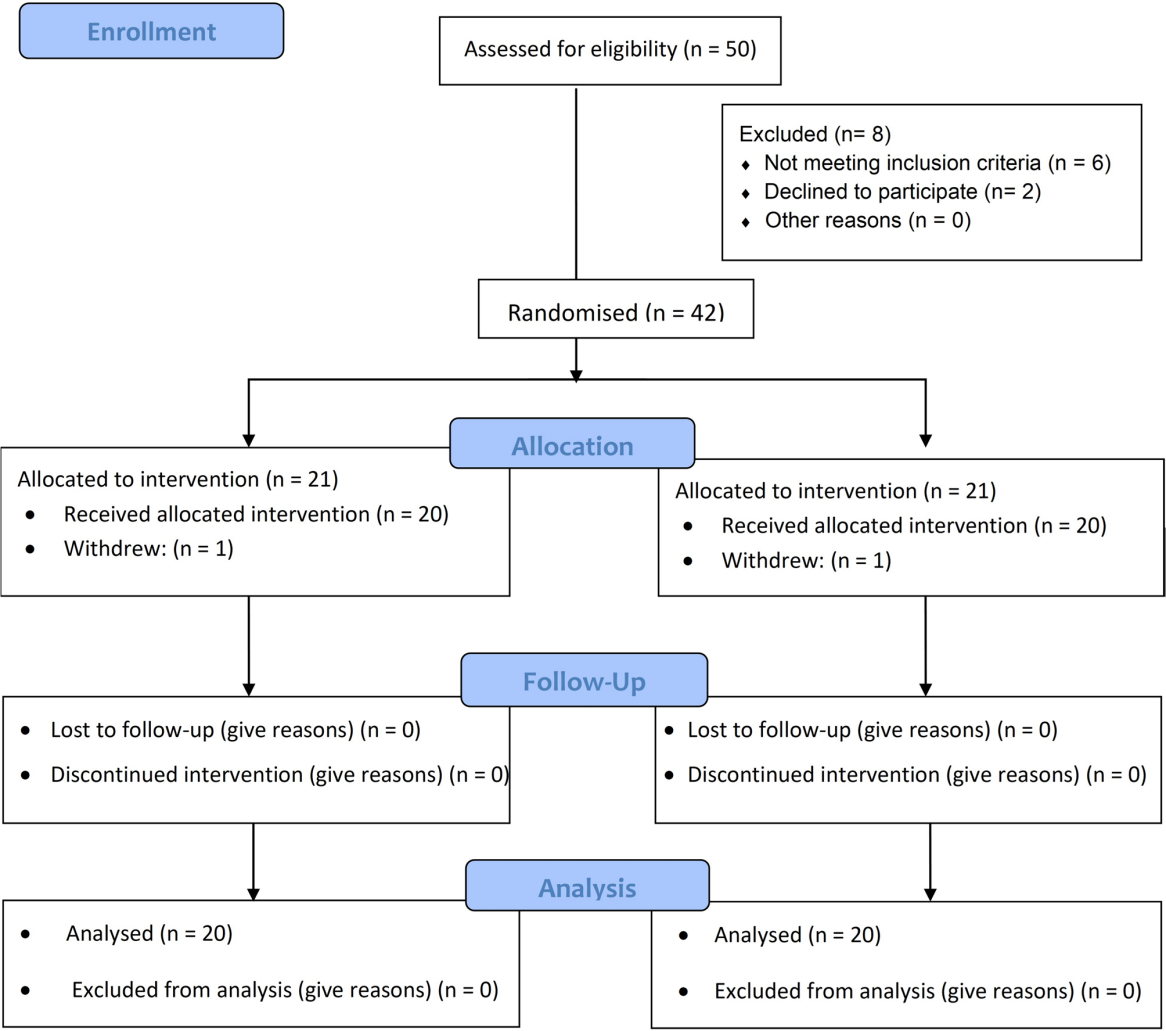
CONSORT flowchart for patient recruitment and analysis

After obtaining informed written consent from every patient, we started patient enrollment in May 2022 and continued until October 2022 in our tertiary university hospital. Forty female patients, American Society of Anesthesiologists (ASA) status I and II, who were scheduled for unilateral modified radical mastectomy (MRM) with axillary lymph node dissection (ALND) under the influence of ESPB and general anesthesia, participated in the current study (Fig. 1). Participants had the right to withdraw consent at any time without facing discrimination.

The exclusion criteria included patient refusal, any contraindication to regional anesthesia, pregnancy, vertebral deformities, prior thoracic spinal procedures, chronic opioid usage, and morbid obesity (Body mass index (BMI) ≥ 40 kg/m^2^).

The sample size was determined using the PASS Version 20 Program to measure the proposed proportional clinically relevant difference in the dermatomal sensory block and the cephalo-caudal spread among both groups (effect size = 25% proportional difference), taking into consideration a 95% confidence level, 80% power, and Significance Level 0.05. The minimum hypothesized total sample size of 40 eligible patients (20 per group) was required [10].

Using a permuted block randomization technique, an online tool was employed to generate the allocation sequence and the block size with a 1:1 ratio. The patient’s allocation and postoperative outcome assessors were blinded, and the same anesthetist (who could not be blinded due to the different patient positions) performed all ESPB procedures. Also, the surgery was performed by the same surgical team. The patients were assigned through opaque envelopes into two equal groups (Fig. 1):

- ➣ Group 1: 20 patients received ESPB in sitting position.
- ➣ Group 2: 20 patients received ESPB in lateral position, with the surgical side being uppermost.

### Measurements

The primary outcome was the ipsilateral dermatomal sensory block from the parasternal line to the scapular line 20 min after the block and radiocontrast material cephalocaudal spread through the erector spinae plane and nearby structures by CT 15 min after the block. The secondary outcomes were the visual analog scale (VAS) for pain intensity, opioid consumption per 24 h, patient satisfaction for block position and analgesia (by Likert Scale), and complications.

In the block room, multiple channels monitor (Dräger vista 120, Drägerwerk AG & Co. KGaA, Germany) was attached to each patient, and a peripheral intravenous line (20-G) was inserted on the contralateral side of surgery. Each patient received fentanyl (20ug) and midazolam (0.05 mg/kg) intravenously as a premedication before the block.

After positioning the patient according to the allocated group, the block was administered completely aseptically following skin disinfection and draping. A high-frequency linear Ultrasound (US) probe (6-13 MHz, SonoSite S-Nerve ™, SonoSite Inc. USA) was applied parasagittally on the back of the ipsilateral surgical side 2 cm lateral to the thoracic vertebra to visualize the T4 transverse process, the trapezius, rhomboid major, and erector spinae muscles (ESM).

We injected 2–3 mL of lidocaine 1% to numb the skin. Subsequently, a 22-G spinal needle was inserted in-plane in the cephalocaudal direction until the tip reached the plane between the transverse process of T4 and the ESM. To confirm the appropriate site of the needle’s tip, 2 mL of normal saline was injected. Following confirmation, we injected 5 mL of radiocontrast material (Omnipaque-300^®^) mixed with 35 mL of 0.25% bupivacaine and 8 mg dexamethasone after negative aspiration. According to the group allocation, the patient was kept in position for 15 min, either in the lateral or high Fowler’s position.

The ipsilateral thoracic sensory block in both groups from T4 upwards and downwards along the parasternal, midclavicular, midaxillary, and scapular lines was assessed every 5 min for 20 min after ESPB using ice cubes. The area of the sensory block was mapped and calculated. A block was considered successful if there was diminished sensation from T2 to T6 on the ipsilateral side of surgery; otherwise, the patient was excluded from the study. A single ultrasound machine and probe were utilized in all cases, and the injection rate and pressure were done manually by the same anesthesiologist and standardized across all procedures, minimizing as much as possible variability in the equipment, technique, and block administration.

**Fig. 2:**
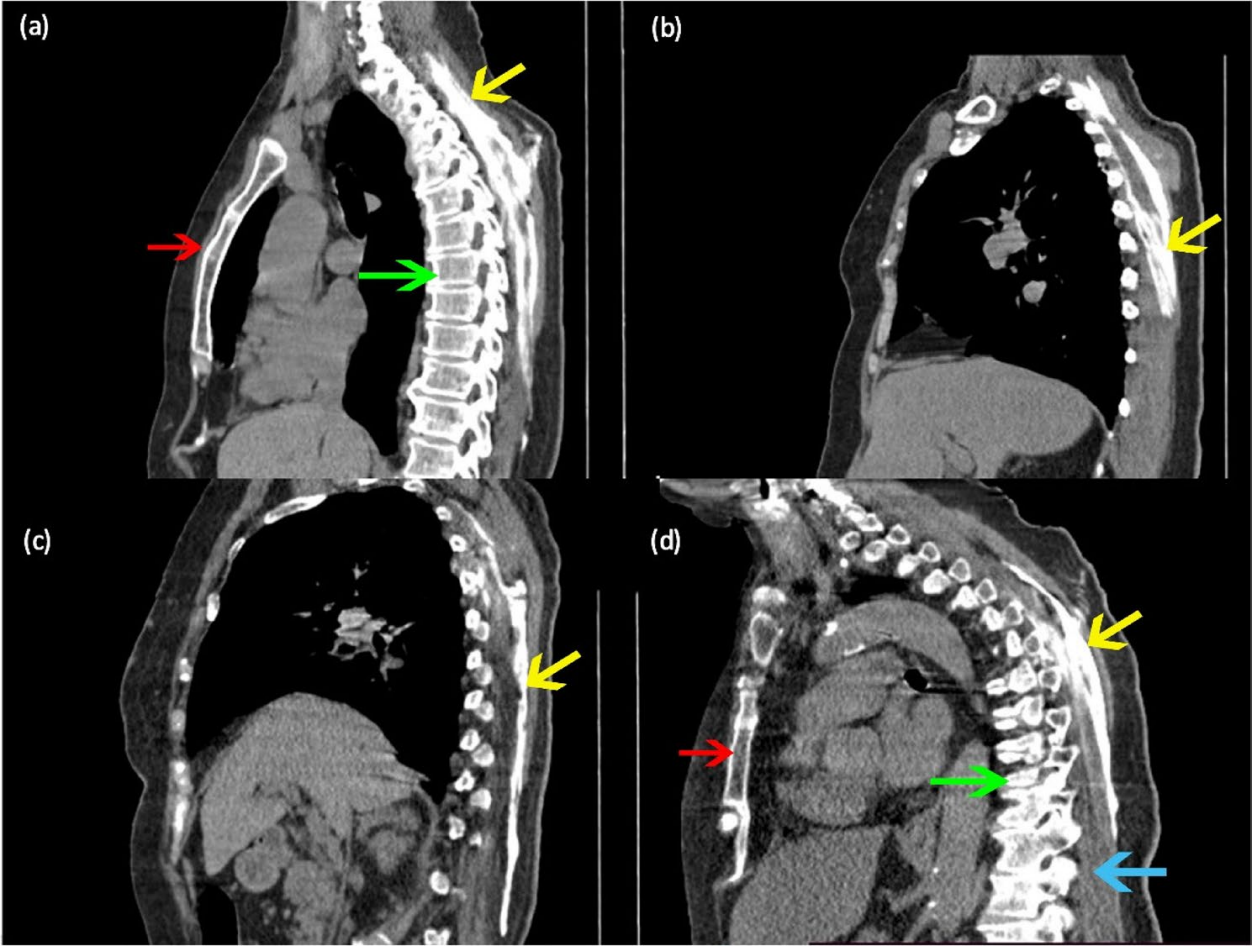
Different sagittal computed tomography cuts (**a**-**d**) of the thoracic region in different patients show retained contrast (yellow arrows) along the erector spinae muscle. Cuts (**a**, **d**) show the manubrium sterni (red arrows), cuts (**a**, **d**) show the dorsal spine (green arrows), and Cut (**d**) shows the erector spinae muscle (blue arrow)

**Fig. 3:**
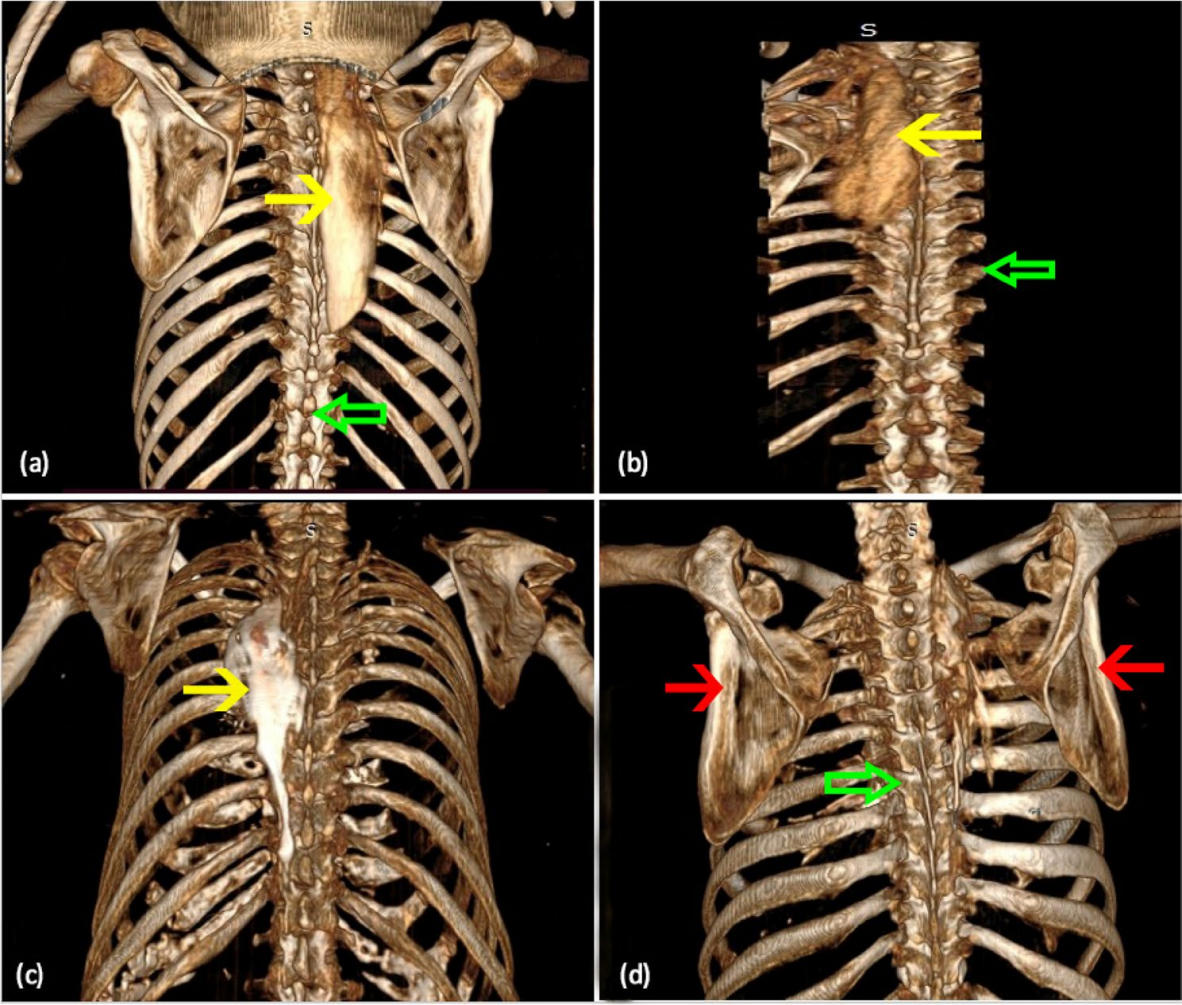
3D reconstruction of the computed tomography imaging of the thoracic region. Cuts (**a**-**c**) showing craniocaudal and lateral contrast spread (yellow arrows) in the thoracic region. Cut (**d**) shows the scapula (red arrows), and cuts (**a**, **b**, **d**) show the dorsal spine (green arrows)

Fifteen minutes following ESPB, a CT scan of the thoracic region was performed. The Digital Imaging and Communications in Medicine (DICOM) program (OsirixX for Mac, PixmeoSARL; Bern, Switzerland) was used to form a 3D digital reconstruction of the distribution of the injected contrast around ESM and adjacent areas, especially paravertebral and epidural spaces (Figs. 2 and 3).

All patients in both groups were transferred to the operating room, where general anesthesia was induced using intravenous fentanyl (1ug/kg), propofol (2 mg/kg), atracurium (0.5 mg/kg) to facilitate tracheal intubation, and isoflurane (1–2%) in a 50%−50% air-oxygen mixture to maintain anesthesia. Fentanyl (25 µg) was administered whenever the heart rate or mean arterial blood pressure increased by 20% or more from the basal levels.

Atracurium (0.1 mg/kg) doses were given as needed to maintain the train of four at two. At the end of the surgery, the residual neuromuscular block was reversed using atropine (0.02 mg/kg) and neostigmine (0.04 mg/kg), and the trachea was extubated.

The VAS of pain at rest was monitored and recorded as soon as the patient arrived from the operating room to the post-anesthesia care unit (PACU). After that, it was recorded every four hours for the following 24 h.

Each patient was attached to an IV morphine patient-controlled analgesia (PCA) pump (Master PCA™, Fresenius vial SA, France) prefilled with a 1 mg/mL concentration of morphine. The PCA’s settings included a 2 mL bolus and a 10-minute lockout without basal infusion. The patient was asked to use PCA on demand if the VAS ≥ 4. The total morphine (mg) consumed over 24 h was calculated.

On the second postoperative day, we assessed the patient’s satisfaction with the ESPB position and the quality of analgesia using the Likert scale (0–4), a self-report scale where 0 = strong dissatisfaction, 1 = dissatisfaction, 2 = neutral, 3 = satisfaction, and 4 = strong satisfaction [11]. Any perioperative complications were recorded, such as and not restricted to (local anesthetic toxicity, vomiting, hypotension, vascular puncture, pneumothorax, and hematoma at the site of injection).

### Statistical analysis

We used IBM SPSS software, version 20.0 (Armonk, NY: IBM Corp.) for the data analysis. We considered results significant at a 5% level and utilized the Shapiro–Wilk test to determine the normality of data distribution. We used the Chi-squared test to compare categorical variables, and when more than 20% of the cells had a count of less than five, we applied Fisher’s exact test to analyze the results. To compare quantitative variables with a normal distribution, we used the student’s t-test to compare between groups. For quantitative variables with abnormal distributions, we employed the Mann-Whitney U test (two-group comparison).

## Results

We started enrollment with 50 patients. Six were excluded because of a surgical decision change. Another two patients declined to participate before the block, and another two declined after receiving the block and refused to complete the study. Demographic data and the side and duration of surgery were comparable between the two studied groups (Table 1). The results showed a significant increase in a dermatomal sensory block in Group 1 in comparison to Group 2 at the midaxillary line (8.45 ± 0.76 vs. 7.90 ± 0.31 sequentially, mean difference (MD) of confidence interval (C.I) 95% = 0.55, *p*-value = 0.006) and at the scapular line (8.45 ± 0.76 vs. 7.95 ± 0.39, sequentially, MD (95% C.I) **=** 0.50, *p*-value = 0.014). Meanwhile, there were non-significant changes between both groups in the parasternal (6.50 ± 1.47 vs. 5.95 ± 1.39 sequentially, MD (95% C.I) **=** 0.60, *p*-value = 0.232) and midclavicular lines (6.50 ± 1.47 vs. 5.90 ± 1.45 sequentially, MD (95% C.I) **=** 0.55, *p*-value = 0.201) (Table 2).

**Table 1:** Demographic data, duration, and side of surgery in the studied groups

Demographic data		Group 1 (20)	Group 2 (20)	*P*
Age (years)		50.70 ± 10.37	57.35 ± 10.49	0.051
BMI (kg/m ^2^)		37.15 ± 1.73	36.40 ± 1.73	0.178
Duration of surgery (min.)		98.45 ± 6.11	98.75 ± 6.86	0.885
Side of surgery	Left	10 (50%)	11 (55%)	0.752
	Right	10 (50%)	9 (45%)	

Cell values represent the mean ± standard deviation (Student t-test) and number of patients (percentage) (Chi-squared test)

p: *p*-value for comparing the two studied groups

**Table 2:** Number of blocked dermatomes after erector spinae plane block in sitting and lateral positions

Dermatomal spread	Group 1 (20)	Group 2 (20)	MD (95% C.I)	*P*
Parasternal line	6.50 ± 1.47	5.95 ± 1.39	0.55	0.232
Midclavicular line	6.50 ± 1.47	5.90 ± 1.45	0.60	0.201
Midaxillary line	8.45 ± 0.76	7.90 ± 0.31	0.55	0.006^*^
Scapular line	8.45 ± 0.76	7.95 ± 0.39	0.50	0.014^*^

Cell values represent the mean ± standard deviation

*p*-value for comparing the two studied groups (Student t-test)

*MD* Mean Difference, *C.I.* Confidence interval

^*^Statistically significant at *p* ≤ 0.05

**Fig. 4:**
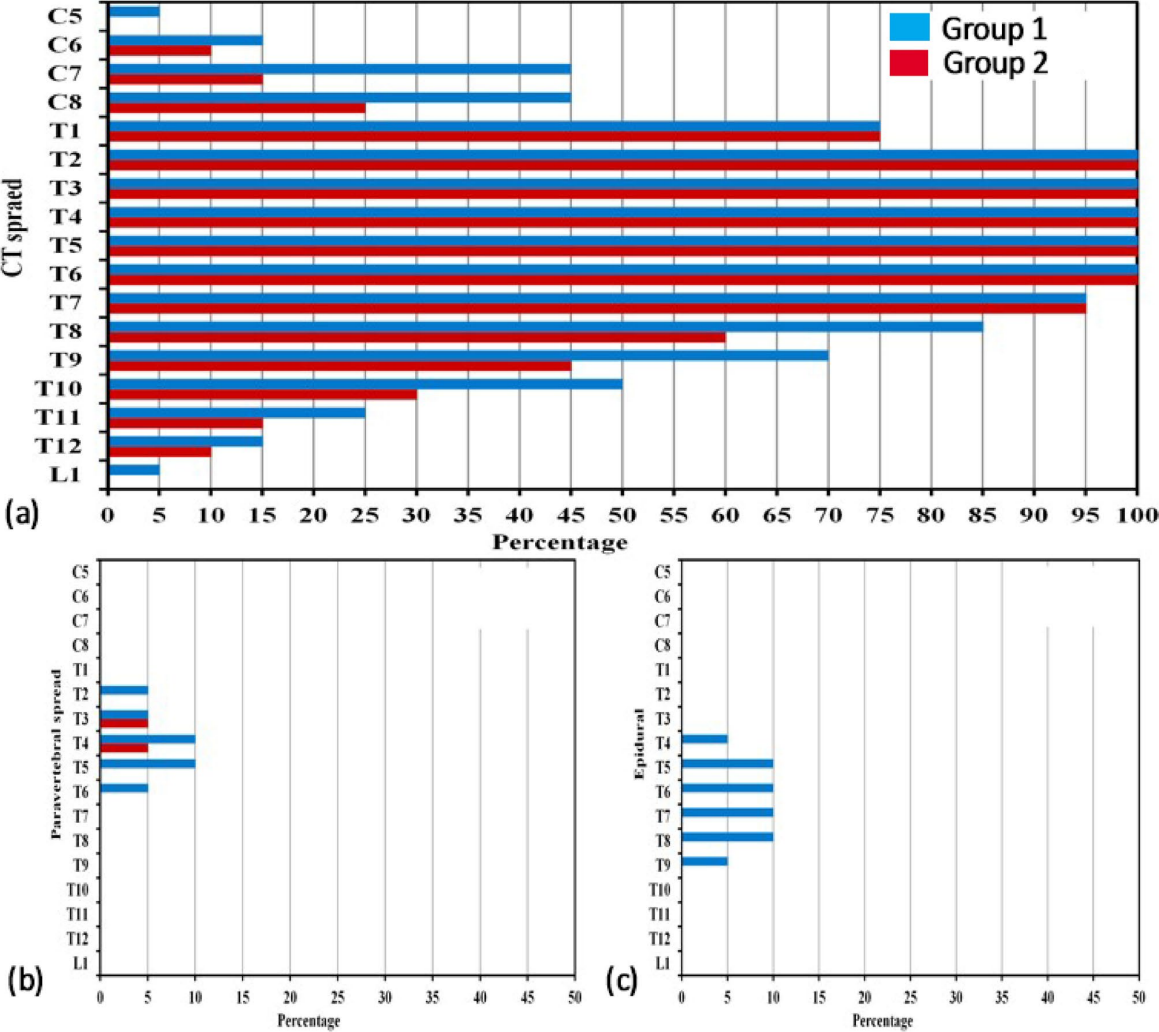
Three bar chart comparisons of CT spread patterns across spinal levels (cervical (C), thoracic (T), and lumbar (L) among groups. It summarizes the craniocaudal (**a**), paravertebral (**b**), and epidural (**c**) contrast spread measured by computed Tomography in both studied groups

We reported more contrast and wider distribution in the craniocaudal direction through the erector spinae plane in Group 1 than in Group 2. However, the difference was statistically non-significant (MD (95% C.I) = 1.05, *p*-value = 0.159) (Table 3). The radiocontrast spread was detected cranially to the cervical (C7) vertebra in 45% of patients in Group 1 compared to 15% in Group 2 and caudally to the thoracic (T10) vertebra in 50% of patients in Group 1 vs. 30% in Group 2. The contrast material extended to the C5 vertebra cranially and caudally to the lumbar (L1) vertebra in 5% and to T12 in 15% of patients in Group 1, while in Group 2, it extended to C6 cranially and T12 caudally in 10% of patients (Fig.  4).

**Table 3:** Percent of patients and segmental vertebral spread of contrast based on computed tomography images in the two studied groups

CT Spread	Group 1 (20)	Group 2 (20)	MD (95% C.I)	*p*
Craniocaudal spread				
(Number of segments)	10.40 ± 2.95	9.35 ± 1.35	1.05	0.159
Paravertebral extension				
Number of patients (%)	6 (30.0%)	2 (10.0%)		^FE^*p*=0.235
(Number of segments)	1.17 ± 0.41	1.0 ± 0.0	0.167	0.604
Epidural spread				
Number of patients (%)	2 (10.0%)	0 (0.0%)		^FE^*p*=0.487
(Number of segments)	5.0 ± 1.41	–		–

Cell values represent the mean ± standard deviation (Student t-test) and number of patients (percentage) (Chi-squared test) *FE* Fisher’s Exact Test

*MD* Mean Difference, *C.I.* Confidence interval

*p*: *p*-value for comparing the two studied groups

**Fig. 5:**
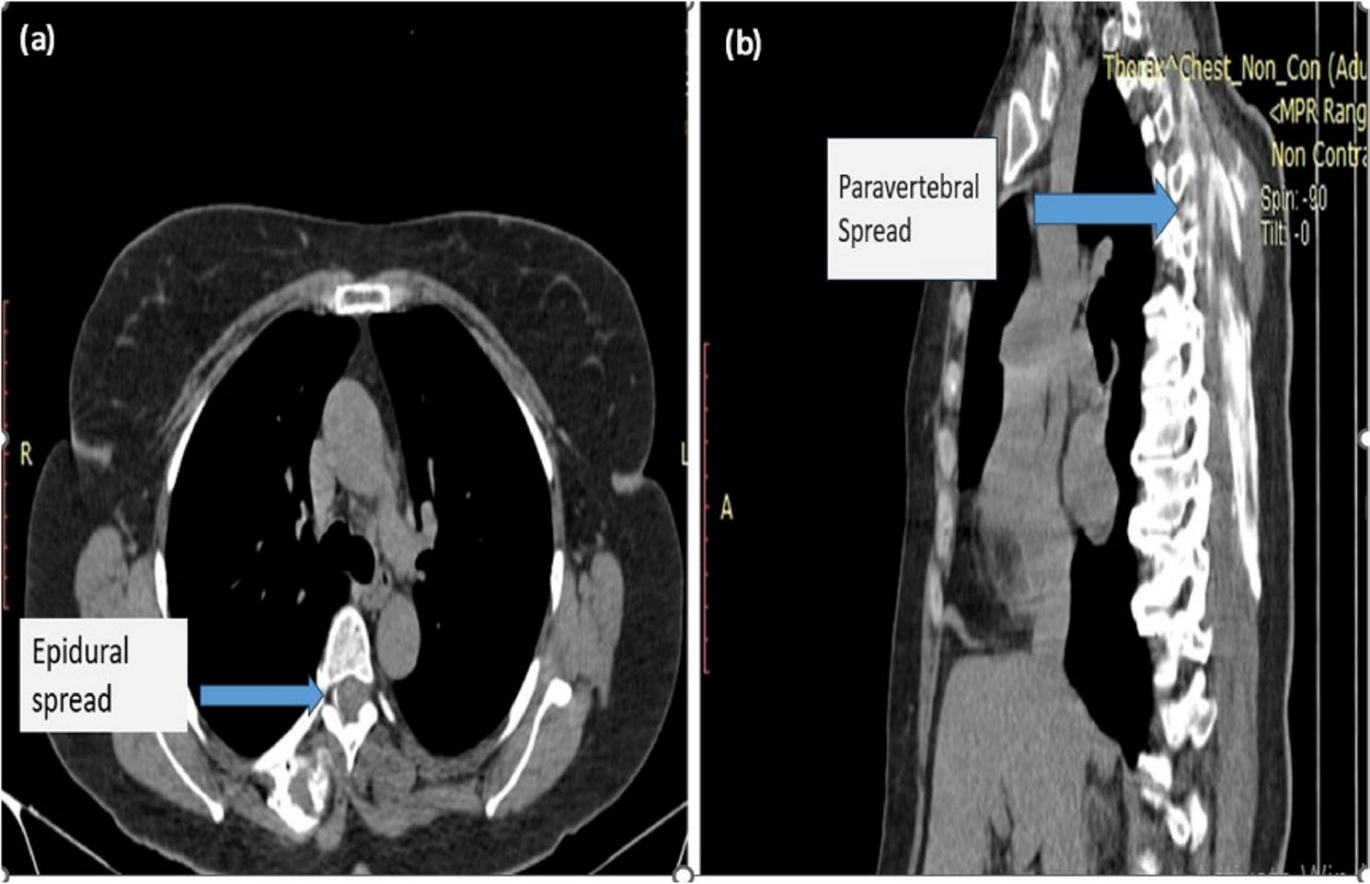
Computed Tomography scan demonstrating the paravertebral and epidural contrast spread after ESPB. (**a**) An axial (transverse) cut of the chest highlights Epidural Spread (A blue arrow). (**b**) A sagittal cut identifies Paravertebral Spread (blue arrow)

**Fig. 6:**
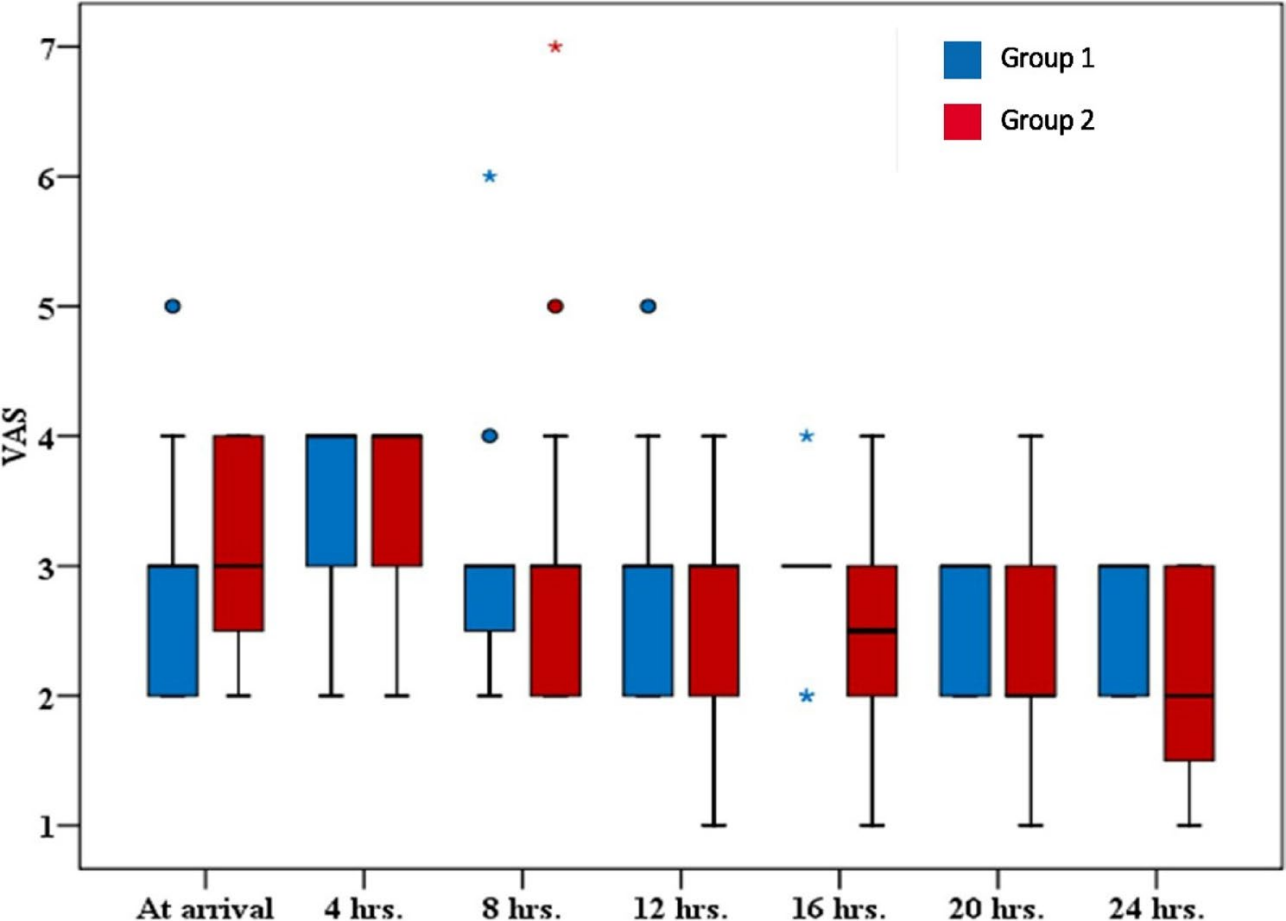
A box plot compares visual analog scale (VAS) scores during rest among groups at various time intervals: upon arrival and at 4-, 8-, 12-, 16-, 20-, and 24-hours postoperative

Thirty percent of patients in Group 1 had dye spread to the paravertebral space (from T2 to T6) vs. 10% in Group 2 (from T3 to T4) and reached epidural space in 5–10% of patients in Group 1 (T4 to T9) vs. no epidural spread in group 2 (Table 3; Figs. 4 and 5). However, differences in paravertebral and epidural dye spread in both groups were non-significant (*p*-value = 0.235 and 0.487, respectively). In the postoperative 24-hour period, we reported no significant variations in the VAS between the studied groups (Fig. 6). The mean value of postoperative morphine consumption (mg) was (2.40 ± 1.23) in Group 1 and (2.0 ± 1.12) in Group 2 (*p*-value = 0.369) (Table 4).

**Table 4:** Total postoperative morphine consumption throughout 24 h after surgery

Total morphine (mg) consumed after 24 h.	Group 1 (20)	Group 2 (20)	*P*
Mean ± SD.	2.40 ± 1.23	2.0 ± 1.12	0.369

*SD* Standard deviation. Cell values represent the mean ± standard deviation

p: *p*-value for comparing the two studied groups (Mann-Whitney test)

Patient satisfaction with position during injection was comparable; 40% of patients were strongly satisfied in Group 1 compared to 55% of patients in Group 2, 45% were satisfied in both groups, and 15% were neutral in Group 1 (Table 5). There were no recorded complications in either group.

**Table 5:** Patient satisfaction with position and quality of analgesia in the studied groups

Patient Satisfaction	Group 1 (20)		Group 2 (20)		*P*
	Number	Percent	Number	Percent	
Position during injection					
Neutral	3	15.0	0	0.0	^MC^p= 0.249
Satisfied	9	45.0	9	45.0	
Strong satisfied	8	40.0	11	55.0	
Postoperative analgesia					
Neutral	0	0.0	0	0.0	0.342
Satisfied	8	40.0	11	55.0	
Strong satisfied	12	60.0	9	45.0	

Cell values represent the number of patients (percentage) (Chi-squared test), *MC* Monte Carlo

p: *p*-value for comparing the two studied groups

Patient satisfaction with postoperative analgesia showed that 60% of patients were strongly satisfied in Group 1 compared to 45% of patients in Group 2, and 40% were satisfied in Group 1 compared to 55% of patients in Group 2 (Table 5).

## Discussion

The ESPB is a convenient regional analgesic technique for thoracic and abdominal surgeries. The mechanism, according to data from clinical and cadaveric studies [12], is direct local anesthetic distribution and dissemination to the nearby neural structures beneath the erector spinae muscles and into the surrounding compartments, including the paravertebral space, epidural space, and nerve roots.

The current trial demonstrated that cutaneous sensory block was significantly more in midaxillary and scapular lines 20 min after ESPB in sitting than in the lateral position and comparable at parasternal and midclavicular lines. The observed differences, though statistically significant, are modest and had limited impact on clinical outcomes (analgesia and opioid consumption). We hypothesized that this might be attributed partially to the fact that we only enrolled patients with successful block coverage of at least dermatomes from T2 to T6 (dermatomal supply of the breast) and the relatively limited sample size.

In consistency with our results, Barrios and his colleagues [13] showed a dermatomal spread of 8–11 dermatomes was reported after a single injection of 20 mL bupivacaine at the mid-thoracic level 60 min after ESPB. The extended duration of sensory assessment may have influenced more dermatomal block due to the slower spread of local anesthetic. Hamilton and Manickam [14] reported a loss of cold sensation (T1-T9), and a partial block of the C7 and C8 dermatomes with ESPB was performed at T5 with a bolus of 20 mL levobupivacaine, followed by a further 15 mL of Levobupivacaine injected via catheter. The volume of the local anesthetic injected could be another factor determining the dermatomal sensory block.

We reported CT evidence of early wider and more contrast distribution in the craniocaudal direction, the paravertebral space, and the epidural spread in a few more patients in sitting positions 15 min after ESPB; however, these differences were mild and not statistically significant. The lack of a full contrast spread profile may be due to the relatively early performance of CT (maybe, if delayed or repeated after surgery, it would demonstrate the full spread pattern).

Also, possible explanations for the earlier craniocaudal expansion of the local anesthetic in the sitting position could be partially attributed to the higher muscle tone of the erector spinae muscle, which might promote a more significant propulsive effect. Viir et al. [15] reported reduced muscle tone when the patient’s position changed from sitting to lying. Furthermore, we postulated that the effect of gravity in the sitting position (for 15 min) and the direction of the injection needle (cephalocaudal) might promote a greater caudal spread and, consequently, the dispersion of the local anesthetic laterally and beyond the transverse processes through the retro superior costotransverse space enhanced by the effect of the hydrostatic pressure of the cephalocaudal fluid column of local anesthetic. These may account for the enhanced incidence of paravertebral and epidural distribution in the sitting position, especially at the injection site (T4) and below it (T5-T9) (Fig. 5) [16, 17]. In contrast, in the lateral decubitus position, the patient is horizontal; anatomically, the gravity would act differently, likely pulling the injectate toward the dependent side (toward the retrolaminar space) or tending to remain around the injection level rather than strictly along the head-to-foot axis.

Multiple cadaveric studies using dissection or radiocontrast imaging proved the distribution of dye craniocaudally [6, 7, 18–28]. Some cadaveric research revealed that it spread to the paravertebral region in a minority of specimens [6, 18, 21–23, 25, 26, 28]. Other studies have denied this [3, 7, 27].

Epidural spread was verified in some cadaveric investigations [18, 19, 21, 23, 25] but was absent in some others [3, 7, 26, 28]. Lateral distribution into the intercostal space may be an explanation for the clinical analgesic efficacy of ESPB. However, CT imaging could not detect the lateral distribution in the present study (poor soft tissue resolution). Magnetic Resonance Imaging (MRI) could be helpful (better soft tissue resolution) in verifying lateral distribution to neural foramina and intercostal nerves. Schwartzmann et al. [29] performed ESPB in six patients at the level of T10 and used a local anesthetic-gadolinium mixture, then performed an MRI for their patients one hour after the block and documented neural foramina and intercostal space spread in all patients.

A recent study was conducted by Sorenstua et al. [30], who performed ESPB on ten volunteers using 30 mL of ropivacaine 0.25% with gadolinium and performed MRI one hour after the block. They reported local anesthetic spread to ESM and intercostal space in all volunteers, paravertebral space in nine volunteers, neural foramina in eight volunteers, and epidural space in only four volunteers.

These studies support ESPB’s analgesic mechanism by dispersing local anesthetic through paravertebral, intercostal, and epidural spaces to nearby nerve roots [17, 29, 30].

In the current study, the ESPB was an effective analgesic modality in both groups. Additionally, there were statistically insignificant variations in postoperative VAS scores and 24-hour intravenous morphine consumption among groups. Similarly, Singh et al. [31] investigated 40 patients undergoing modified radical mastectomy (MRM) and reported a reduction in morphine consumption over 24 h in ESPB compared to sole general anesthesia.

A recent meta-analysis by Cui et al. [32] included 52 studies with 3000 patients undergoing various surgical procedures (breast surgeries, orthopedic surgeries, thoracic surgeries, cardiac surgeries, nephrolithotomy, and cholecystectomy). They demonstrated that ESPB minimized the total opioid consumption in the first 24 postoperative hours and increased the time between surgery and the first rescue analgesia.

With the minimal higher preference of patients for the lateral position, the current study showed a non-significant difference in both groups’ patient satisfaction with block position and analgesia. Consistent with these findings, Park et al. [33] demonstrated that ESPB improved patient satisfaction with analgesia after mastectomy. Furthermore, Yao et al. [34] and Oh and his colleagues [35] showed that ESPB greatly enhanced patient satisfaction and the quality of recovery.

A significant advantage of the present study was examining the ESPB spread in living patients in two distinct positions and evaluating the spread clinically and radiologically (CT imaging). Although the sitting group was associated with greater early contrast spread (and possibly onset) and a higher incidence of paravertebral/epidural spread, this did not result in lower VAS scores, reduced morphine use, or higher statistically significant patient satisfaction (may be partially attributed to that we only enrolled patients with successful block covering at least dermatomes from T2 to T6). The lateral position’s comfort and convenience during the block could make it preferable in certain clinical situations (especially those in pain or with limited mobility), as the patient can remain lying down rather than sitting upright.

The study has opened new questions and areas for exploration regarding the effect of the position during variable regional blocks on the efficacy of analgesia to provide the optimum position during the block, offering the maximum spread and satisfaction for both patients and anesthetists and thus improving perioperative analgesia and care.

The study has limitations, including being a single-center study, and larger multi-center studies need to be conducted, relying on MRI or repeated CT scans to provide stronger evidence regarding the difference in LA spread position and effect on postoperative analgesia. The VAS was measured at rest only, and future studies should incorporate dynamic pain measurements and chronic pain assessment. Also, the CT was done once and shortly after the block (our use of CT was based on practical and ethical considerations of availability, limiting radiation exposure, and the need for a quick, feasible imaging option without causing a significant delay in the operative schedule). While early CT is informative about initial spread, it does not tell the full story of the extent and pattern of spread– hence, both early and late CT and MRI are useful in future research.

## Conclusion

The Erector Spinae Plane Block in the sitting position provided a more significant posterolateral sensory block than in the lateral position. The CT evidence of early cephalocaudal, paravertebral, and epidural spread of local anesthetic was higher in a minority of patients in sitting positions and was statistically nonsignificant. ESPB in both patient positions produced comparable effective postoperative opioid consumption for mastectomy.

## Data Availability

The data related to this study is available from the corresponding author upon reasonable request.
